# Genome-wide identification of the PYL gene family of tea plants (*Camellia sinensis*) revealed its expression profiles under different stress and tissues

**DOI:** 10.1186/s12864-023-09464-5

**Published:** 2023-06-28

**Authors:** Yanlin An, Xiaozeng Mi, Xiaobo Xia, Dahe Qiao, Shirui Yu, Huayan Zheng, Tingting Jing, Feng Zhang

**Affiliations:** 1 Department of Food Science and Engineering, Moutai Institute, Luban Street, Renhuai, 564502 Guizhou P.R. China; 2 Tea Research Institute, Guizhou Academy of Agricultural Sciences, 1 Jinxin Community, Guiyang, 550025 Guizhou China; 3 College of Plant Protection, Nanjing Agricultural University, Nanjing, 210095 China; 4 State Key Laboratory of Tea Plant Biology and Utilization, Anhui Agricultural University, 130 Changjiang West Road, Hefei, China

**Keywords:** PYL gene family, Tea plant, Gene expression, Stress, Tissue

## Abstract

**Background:**

PYL (Pyrabactin resistance 1-like) protein is a receptor of abscisic acid (ABA), which plays an important role in ABA signaling and influences plant growth and development and stress response. However, studies on PYL gene family in tea plants have not been reported.

**Results:**

In this study, we identified 20 PYL genes from the reference genome of tea plant (‘Shuchazao’). Phylogeny analysis indicated that PYLs from tea and other plant species were clustered into seven groups. The promoter region of PYL genes contains a large number of cis-elements related to hormones and stresses. A large number of PYL genes responding to stress were found by analyzing the expression levels of abiotic stress and biotic stress transcriptome data. For example, CSS0047272.1 were up-regulated by drought stress, and CSS0027597.1 could respond to both anthracnose disease and geometrid feeding treatments. In addition, 10 PYL genes related to growth and development were verified by RT-qPCR and their tissue expression characteristics were revealed.

**Conclusions:**

Our results provided a comprehensive characteristic of the PYL gene family in tea plants and provided an important clue for further exploring its functions in the growth and development, and resistance to stress of tea plants.

**Supplementary Information:**

The online version contains supplementary material available at 10.1186/s12864-023-09464-5.

## Background

Tea plant (*Camellia sinensis* (L.) O. Kuntze) is an important woody crop of significant economic, health, and cultural importance [1]. Tea beverages made from the leaves of tea plant is one of the three most popular nonalcoholic beverages consumed worldwide [2]. Due to the unique flavor and quality characteristics of tea, a large number of studies have focused on its growth and development and metabolites [3, 4]. For example, analysis of theanine metabolic pathway based on transcriptome sequencing has revealed its synthesis mechanism in different tea plant varieties and tissues [5]. In addition, glutamate dehydrogenase GDH2 can negatively regulate theanine accumulation of tea shoots in late spring [6]. A large number of alternative splicing events related to flavonoid metabolism were identified through the identification of alternative splicing in different tissues of tea plants [7]. Tea plants are perennial evergreen crops and live in a relatively stable ecological environment, which makes it more vulnerable to some biotic and abiotic stresses [8, 9]. Zhu et al. identified lipoxygenase (LOX) family genes and their alternative splicing events, revealing their important role in the resistance of tea plants to low temperature, pathogen infection and feeding by tea geometrid [10]. Under drought stress, exogenous abscisic acid can cause the resistance to drought due to the change of its proteome in tea plant [11]. Therefore, it is of great significance for the key regulatory genes and signal transduction mechanisms affecting the growth and development and the response to stress of tea plants.

Abscisic acid (ABA) is the hormone that plays an important role in plant growth and development and in resistance to various stresses [12]. For example, many studies have demonstrated that ABA promotes leaf senescence and maintenance of seed dormancy [13, 14]. In addition, stresses also affect ABA homeostasis. Under drought stress, the content of ABA will increase, and the increase of ABA content can close the open stomata through the regulation of phospholipase (*PLDα1*) to prevent water loss [15]. Mainly, ABA is known as a signaling molecule and triggers the signal transduction [16, 17]. In the process of ABA signal reception and transmission, PYRABACTIN RESISTANCE PROTEINS/PYR-LIKE PROTEINS/REGULATORY COMPONENTS OF ABA RECEPTOR (PYR/PYL/RCAR) receptors, PHOSPHATASE 2 C (PP2C) phosphatases, and SNF1-RELATED PROTEIN KINASE2 (SnRK2) kinases are required to participate [18–20]. Among them, ABA receptor is the upstream regulator of ABA pathway and plays an important role in the regulation of ABA signal.

**Fig. 1:**
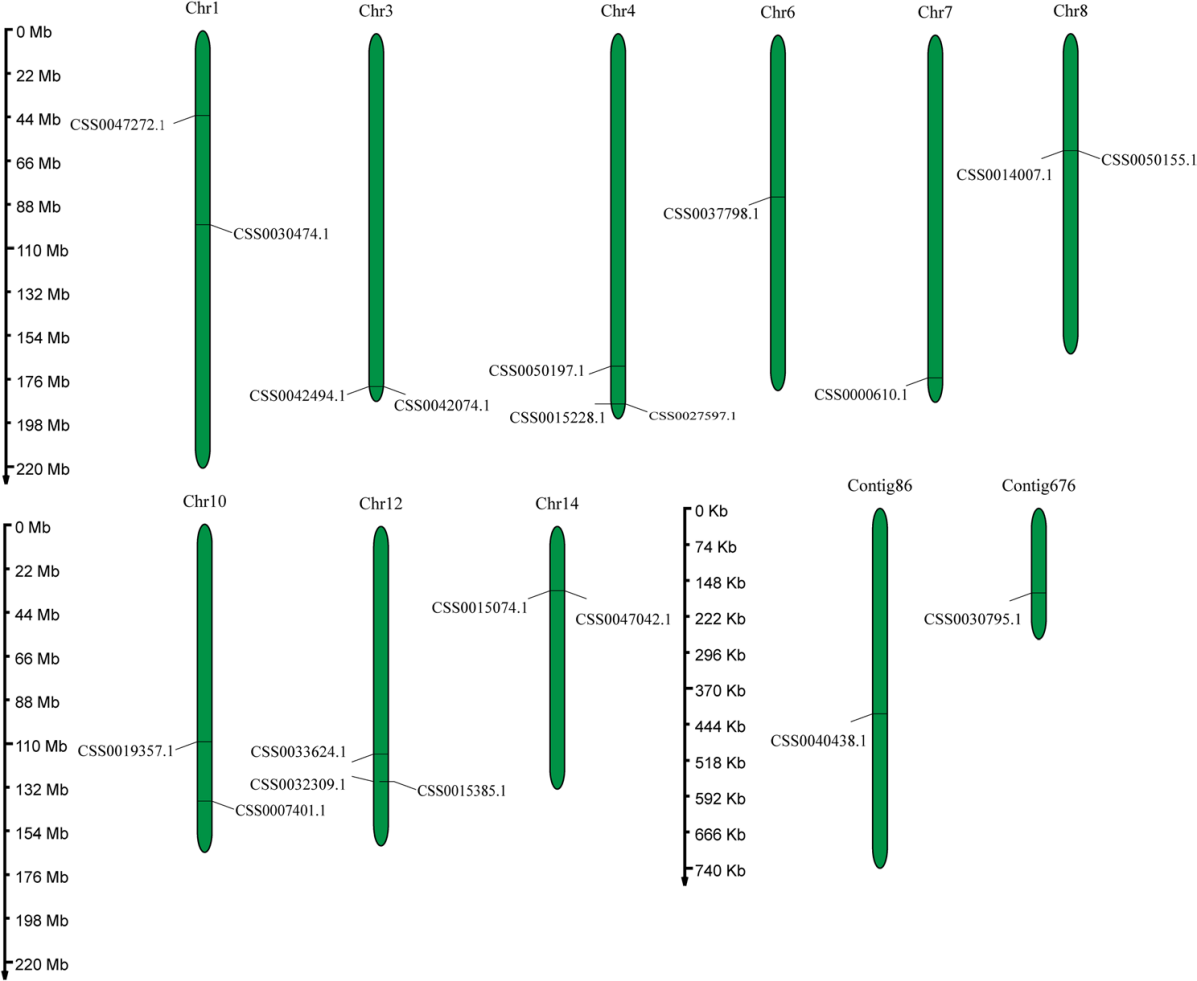
Distribution of the PYL genes in the ‘Shuchazao’ genome. The left axis shows the length of each chromosome and contig, and it was estimated in mega base (Mb)

Pyrabatin Riesistance 1-Like (PYL) protein is the receptor protein of ABA, and an important component of its core signal pathway PYLs-PP2Cs-SnRK2 [21]. The ABA receptor PYL protein was discovered at 2009 by Sean Cutler through chemical genetic screens for mutants that are insensitive to the ABA analog pyrabactin [22]. Studies have been found that ABA can bind to PYL protein to change their conformation, and then it forms PYL-ABA-PP2C complex with PP2C, which inhibits the activity of PP2C, thus releases the protein kinase SnRK2 that binds to PP2C [23]. The activated SnRK2 phosphorylates downstream transcription factors and ion channels to activate ABA signaling pathways and stress response processes [24]. PYL protein containing the START (the star-related lipid-transfer domain) characteristic domain and belongs to the START protein superfamily. Their sequence is conserved and contains cavities binding hydrophobic ligands [25]. At present, PYL family genes have been identified in many plants according to its conserved gene sequence. In Arabidopsis thaliana, there are 14 members of the PYL family, including 13 ABA receptors and one non-responsive PP2C regulator PYL13 [26, 27]. Furthermore, 13, 11, and 27 PYL genes were also identified in rice [28], cucumber [29], and cotton [30], respectively. As a core component of ABA signaling, numerous reports have shown that PYL genes play an important role in growth and development and response to stress in plants [31, 32]. The expression analysis of 11 ABA-related rate-limiting enzyme genes in different tissues of grape showed that *VvPYL1* was tissue-specific and the expression level was the highest in roots, then overexpression of *VvPYL1* in Arabidopsis thaliana was found to be longer in root hairs than that in wild-type plants [33]. In wheat, overexpression of *TaPYL4* gene enhances drought resistance by reducing transpiration and improving water use efficiency [34]. In addition, *ZmPYL8*, *ZmPYL9* and *ZmPYL12* plays an important role in the process of drought resistance of maize [35]. However, the function of PYL gene in tea plant growth and development and stress has not been reported.

Here, we comprehensively identified the members of PYL gene family in tea plant, and analyzed its structure, motif, cis-element and synteny through its sequence characteristics. In addition, abundant transcriptome data were used to analyze the expression patterns of PYL gene under different abiotic and biotic stresses, and multiple PYL candidate genes responding to stress were found. Through quantitative analysis of different tissues, the role of PYL gene in the growth and development of tea tree was revealed. This study will provide a global perspective to study the evolution and functional characteristics of the PYL genes family in tea plants, and also provided a evidence for further research on the role of PYL gene family in the growth and development and resistance to stresses.

## Materials and methods

### Plant materials

*C. sinensis* cv. Shuchazao (a clonal shrub-type and middle-leaf variety selected from the local varieties in Shucheng county) were grown in the Germplasm Tea Repository of Guizhou Tea Research Institute located in Guiyang (N26^°^30’, E106^°^39’), Guizhou Province, China. The tissues sampled were taken as follows: bud, first leaf, second leaf, third leaf, fruits, stems, flowers and roots (the plants of the picked samples are in a natural growth state). All samples contained three biological replicates, and all collected samples were immediately frozen in liquid nitrogen. The sample is then stored at -80^°^ C before use.

### Identification of the PYL family in tea plant

The genome of the tea plant (*Camellia sinensis* var. sinensis cv. ‘Shuchazao’, SCZ) was used as the reference genome [36], and whole genome sequences were downloaded from the TPIA database (http://tpia.teaplant.org/) [37]. To identify the number of PYLs family in tea plants, protein sequences of PYLs in *Arabidopsis* were obtained from TAIR (https://www.arabidopsis.org/) database. Homologous alignment was made between the PYL gene family protein sequence of *Arabidopsis thaliana* and the whole genome protein sequence of tea plant, and the protein sequence with more than 75% alignment similarity was retained. In addition, the Pfam sequence containing the PYL domain was collected from the Pfam database (http://pfam.sanger.ac.uk/) by PF10604.11 [38]. Subsequently, Hidden Markov Model (HMM) was employed to identify PYL genes in tea plant using hmmsearch (v3.3.2) software with Pfam sequence [39]. The sequences with homologous alignment and HMM (e-value < 1e^− 20^) were considered as candidate PYL family. The candidate sequences were further screened by searching for the PYL domain using PFAM (http://pfam.xfam.org/) and SMART (http://smart.embl-heidelberg.de/). Finally, the sequences that pass both databases were considered as PYLs family.

### Chromosome distribution and synteny analysis

MG2C website (http://mg2c.iask.in/mg2c_v2.1/) was used to visualized the distribution of all PYL genes on chromosome with the GFF file. For the synteny analysis within the genome, blast (v 2.12.0+) was used to compare the sequence of PYL proteins with the reference protein sequence [40]. Then, gene synteny analysis were conducted with MCScanX software, and diagrams were generated using TBtools (v1.108) software [41, 42]. The genome sequences of wild tea tree (DASZ) and *Camellia chekiangoleosa* (HHYC) were obtained from the National Center for Biotechnology Information (NCBI, https://www.ncbi.nlm.nih.gov/) and the China National Center for Bioinformation (https://ngdc.cncb.ac.cn/gwh), with accession number PRJNA595851 and GWHBGBN00000000, respectively [43, 44]. Analysis of gene synteny between, SCZ, DASZ, and HHYC were conducted with MCScanX software using the amino acid sequences and chromosome position data, and also visualized by Tbtools software.

**Fig. 2:**
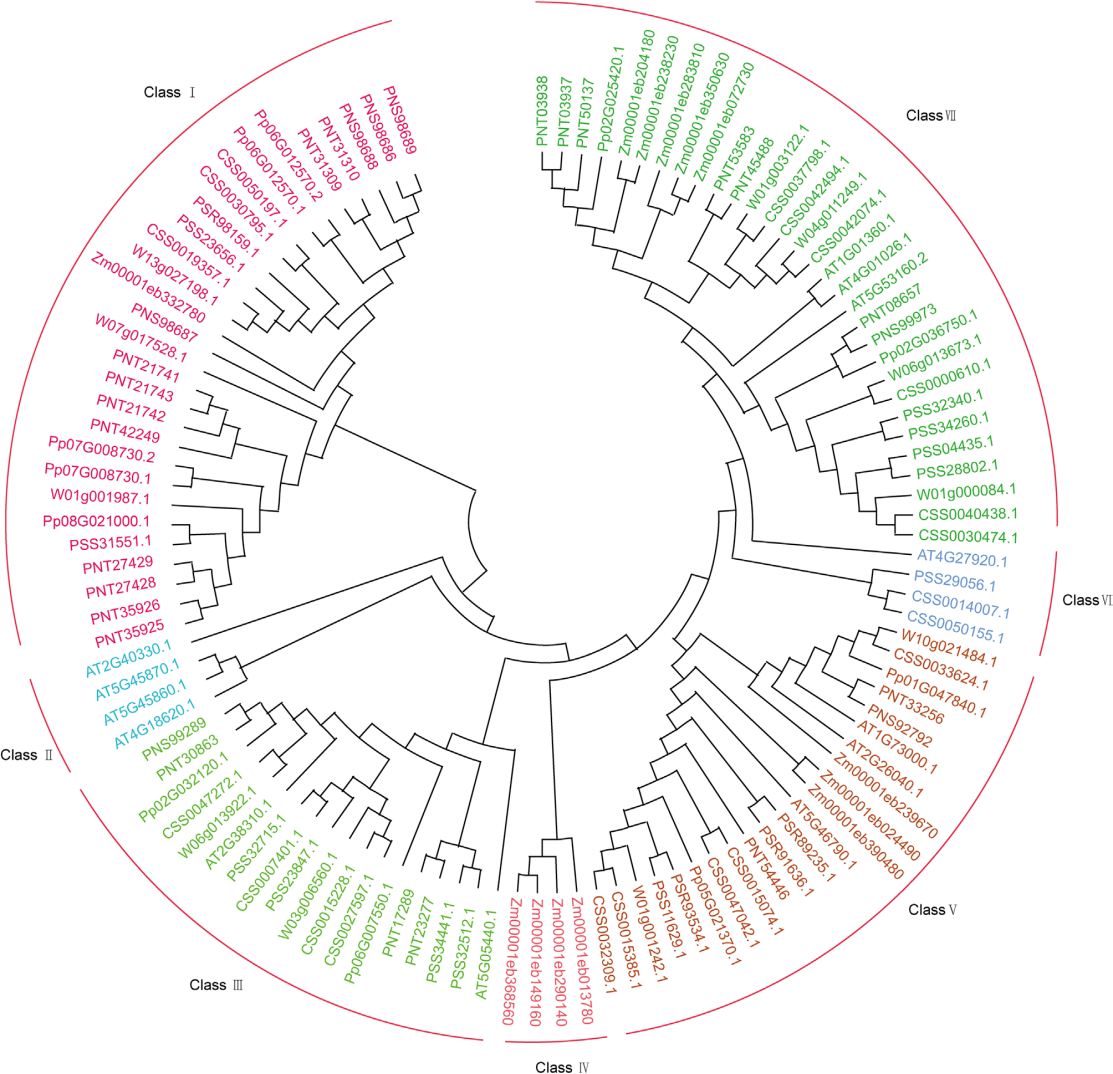
Phylogenetic analysis of PYL genes in tea plant. An unrooted Neighbor–Joining phylogenetic tree was constructed from *Arabidopsis*, Maize, Kiwifruit, Poplar, Peach, SCZ and DASZ. The bootstrap test was set to 1,000 replicates. Different colors represent different subfamilies of the PYL gene

### Phylogenetic tree construction

Protein sequences of *Arabidopsis thaliana* (https://www.arabidopsis.org/), maize (https://maizegdb.org/), kiwifruit (http://kiwifruitgenome.org/), poplar (https://phytozome-next.jgi.doe.gov/), peach tree (http://www.stylebio.cn/index.html) and wild tea (DASZ) were downloaded and used for identification and screening of PYL gene family members. Multiple sequences alignment analysis was carried out using the ClustalW program built in MEGA11 software [45]. The phylogenetic tree was constructed using the NJ (Neighbor–Joining) method with the following parameters: Poisson model, bootstrap method = 1,000 replicates, Uniform rates, Pairwise deletion. PYLs family was classified according to clustering.

### Sequence analysis of PYL family

Coding sequences (CDS), protein sequences and GFF files of PYLs genes were obtained from the reference genome for subsequent analysis. The molecular weight (MW) and isoelectric point (PI) of all PYLs proteins were predicted using the ExPasy web site (https://web.expasy.org/). Motif analysis of the protein sequences were conducted on the MEME website (http://meme-suite.org/), and parameters of the motifs were set as 10. Cis-acting regulatory elements were annotated using PlantCARE online tool (http://bioinformatics.psb.ugent.be/webtools/plantcare/html/). The prediction results of motif and Cis-acting regulatory elements were visualized by TBtools software [46]. The gene structure of intron and exon was visualized by the TBtools software through the GFF file of the genome annotation.

### Transcriptomic data analysis

The SRAToolkit (v 3.0.0) was used to download RNA-seq datasets from the Sequence Read Archive (SRA) database (https://www.ncbi.nlm.nih.gov/sra/) with their accession number. Detailed information of transcriptome data was shown in Table S1. Trimmomatic (v0.36) software was used to filter the raw data of RNA-seq according to the following key parameters: SLIDINGWINDOW:5:20 LEADING:5 TRAILING:5 MINLEN:50 [47]. The filtered RNA-seq reads were mapped to the reference genome using Hisat2 (v2.1.0) software with default parameters [48]. The samtools (v1.13) software was used to convert the SAM file from mapped to the reference genome into a BAM file [49]. Then, transcripts were assembled using StringTie software (v.1.3.5) with default settings, and transcripts per kilobase million (TPM) was calculated to estimate gene expression levels [48]. The TPM values of all PYL genes were screened, and heat map of their expression levels were visualized by TBtools software [42].

### RNA extraction and quantitative real-time PCR (RT-qPCR)

Total RNA was extracted from all samples using the Total RNA Purification Kit (cat DP441, Tiangen, China) according to the manufacturer’s protocol. The quality and quantity of the RNA extract was determined using agarose gel electrophoresis and the Nanodrop 2500 (Thermo Fisher Scientific, US). First-strand cDNA was synthesized using the PrimeScript RT Reagent Kit (cat RR036A, Takara, Japan) with the manufacturer’s protocols. The RT-qPCR was performed using 10 ul PCR products, and the circulating conditions were referred to our previous study [50]. The relative gene expression values were analyzed using the 2^−ΔCt^ method (each sample has three biological replicates, and each biological replicate includes three technical replicates). All reactions were run in technical triplicates for each sample. The *CsGAPDH* gene was selected as the internal control (GenBank: GE651107) [51]. In order to understand the amplification efficiency of the RT-qPCR primers, and five pairs of primers were randomly selected to analyze the amplification efficiency. The results showed that the amplification efficiency was between 95% and 105%. The relevant primers are listed in Table S2.

**Fig. 3:**
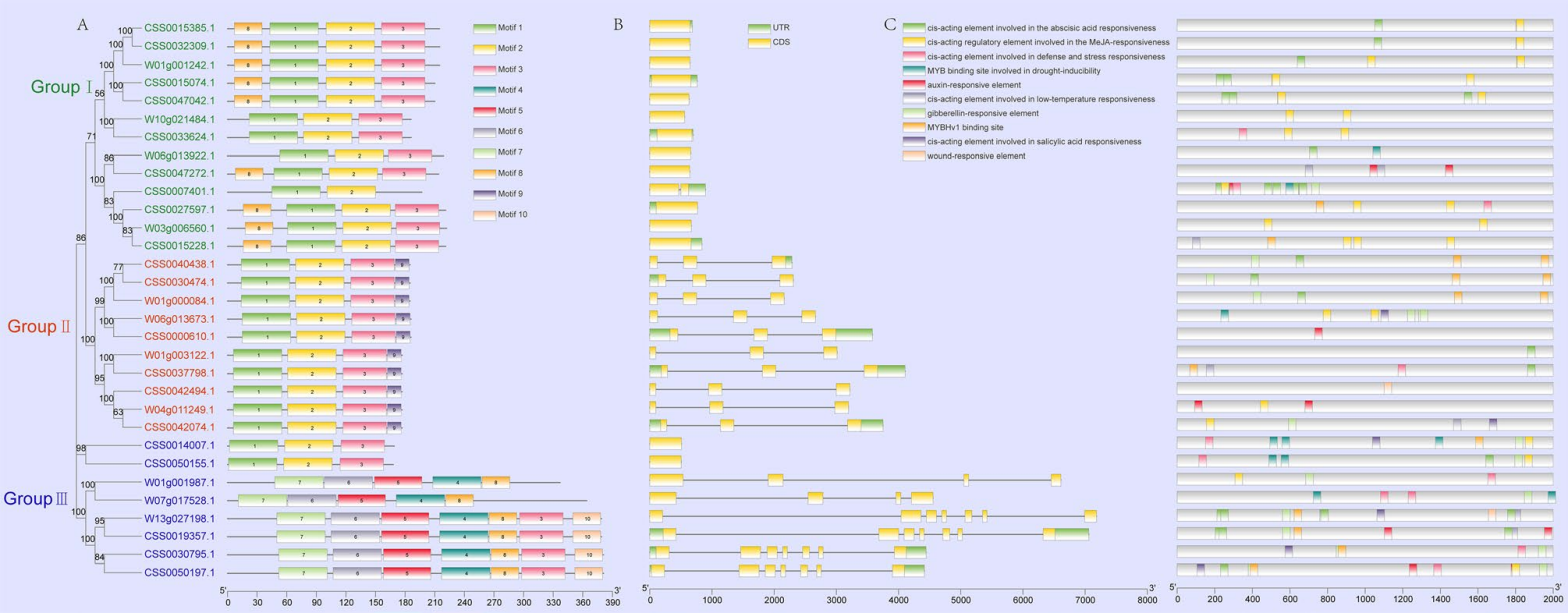
Motif, gene structure and cis-acting regulatory elements analysis of the PYL gene family. (A) Protein motifs of PYL genes. Ten conservative motifs are shown in the figure. (B) Gene structure of PYL genes. The yellow and green blocks indicate exons and UTR respectively, and the lines represent introns. The lengths of blocks and lines represent relative sequence lengths. (C) Cis-acting regulatory elements analysis of the PYL genes. Sequences of the 2000 bp above the start codon were used to identify cis-acting elements

## Results

### Genome-wide identification of the PYL gene family

A total of 20 PYL genes were identified in the ‘Shuchazao’ genome based on HMM model. Except CSS0040438.1 and CSS0030795.1 located in contig 86 and contig 676, all other PYL genes are unevenly distributed on 9 chromosomes. Among them, chromosomes 6 and 12 each contain three PYL genes, and other chromosomes contain two PYL genes (Fig. 1). The main characteristics of PYL genes were analyzed (Table S3). The CDS lengths of PYL genes ranged from 507 bp (CSS0050155.1) to 1146 bp (CSS0030795.1 and CSS0050197.1). The length of proteins ranged from 168 aa (CSS0050155.1) to 381aa (CSS0030795.1 and CSS0050197.1). The MW ranged from 18.87 kDa (CSS0050155.1) to 41.94 kDa (CSS0050197.1), and pI range from 4.96 (CSS0032309.1 and CSS0015385.1) to 7.72 (CSS0042494.1 and CSS0007401.1). Sequence analysis showed that the sequence of some genes in PYL family was highly homologous and had the same CDS, amino acid number and MW, such as CSS0040438.1 and CSS0030474.1. This result suggested that they might have collinearity within the genome. In addition, the same method was used to identify PYL genes in the genome of DASZ, and found that there were only 11 PYL genes, indicating that PYLs have undergone expansion in cultivated tea plant.

**Fig. 4:**
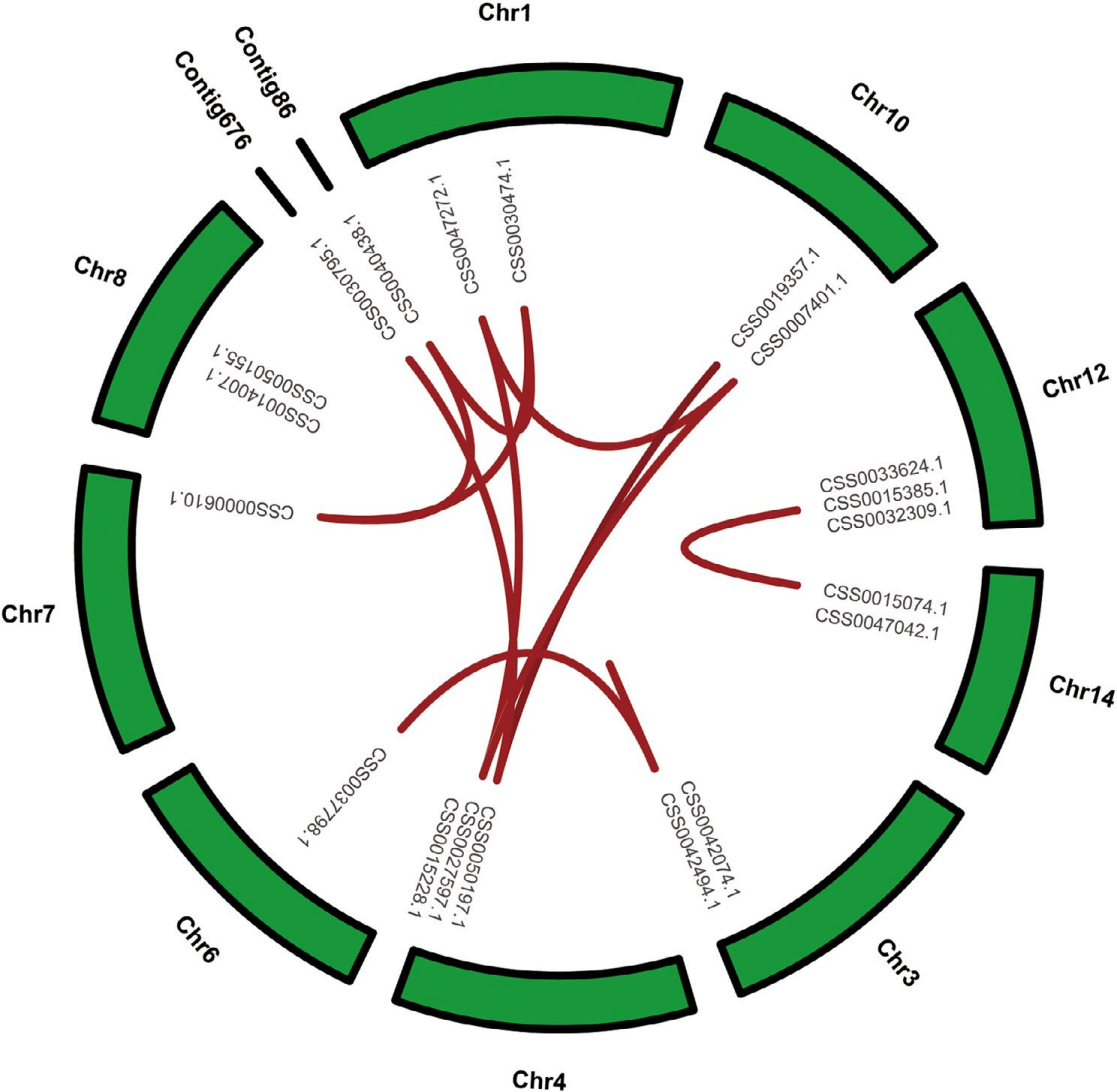
Circle plot showing collinearity of PYL gene family in ‘Shuchazao’ Collinearity genes were highlighted with red curved lines

### Phylogenetic analysis of the PYL gene family

To investigate the evolutionary relationship of PYL genes, a total of 31 PYL amino acid sequences from SCZ and DASZ constructed a phylogenetic tree together with *Arabidopsis*, poplar, peach, maize and kiwifruit (Fig. 2). All sequences are divided into seven subfamilies based on their sequence similarities. Among them, class I contains 3 PYL genes of SCZ and DASZ, while class III contains 4 PYL genes of SCZ and 2 PYL genes of DASZ, respectively. For class V and class VII, there are 5 and 6 SCZ PYL genes respectively. Interestingly, only 2 PYL genes of SCZ were included in class VI, while not included in DASZ, suggesting that the function of PYL genes may be different between the two tea plants.

### Motif, gene structure and cis-acting regulatory elements analysis of the PYL gene family

A total of 31 PYL genes from cultivated and wild tea plants were divided into group I, group II and group III, and each group contained 13, 10 and 8 PYL genes respectively. (Fig. 3A). To reveal the internal relationship of the PYL gene family, conserved motifs of SCZ and DASZ were predicted by MEME. The results show that the members of Group I and Group II all contain Motif 1, Motif 2 and Motif 3, while motif 9 only exists in Group II. Gene structure analysis showed that the number of exons in PYL genes family of tea plants were 1, 3, 4 and 7, respectively. Among them, the exon with the least number was 1, such as CSS0014007.1 and CSS0015385.1. The maximum exon number of w13g0271981, CSS0030795.1, CSS0050197.1 and CSS0019357.1 genes is 7 (Fig. 3B).

To better understand the regulatory network of PYL genes, we analyzed the promoter regions of PYL genes. Two thousand upstream sequences of PYL genes were collected as their promoter, and cis-acting elements were identified by PlantCARE online website. We found 5 types of cis-elements related to hormone of abscisic acid (ABA), methyl jasmonate (MEJA), salicylic acid (SA), gibberellin, and auxin (Table S4). Among the hormone-related elements, there were nine ABA responsiveness elements in the CSS0007401.1 gene, indicating that CSS0007401.1 plays an essential role in ABA response (Fig. 3C). In addition, a total of 13 and 5 PYL genes in SCZ and DASZ were found involved in salicylic acid (SA) and MeJA response, such as CSS0015074.1 and W06g013673.1. In addition, we also identified many stress-related action elements in the promoter region of PYL gene, such as low temperature response elements, wound response elements, defense and stress response elements. For example, two cold-related elements were found in the promoter region of the CSS0047272.1 gene. These results suggesting that the PYL genes play an important role in the abiotic stress resistance.

**Fig. 5:**
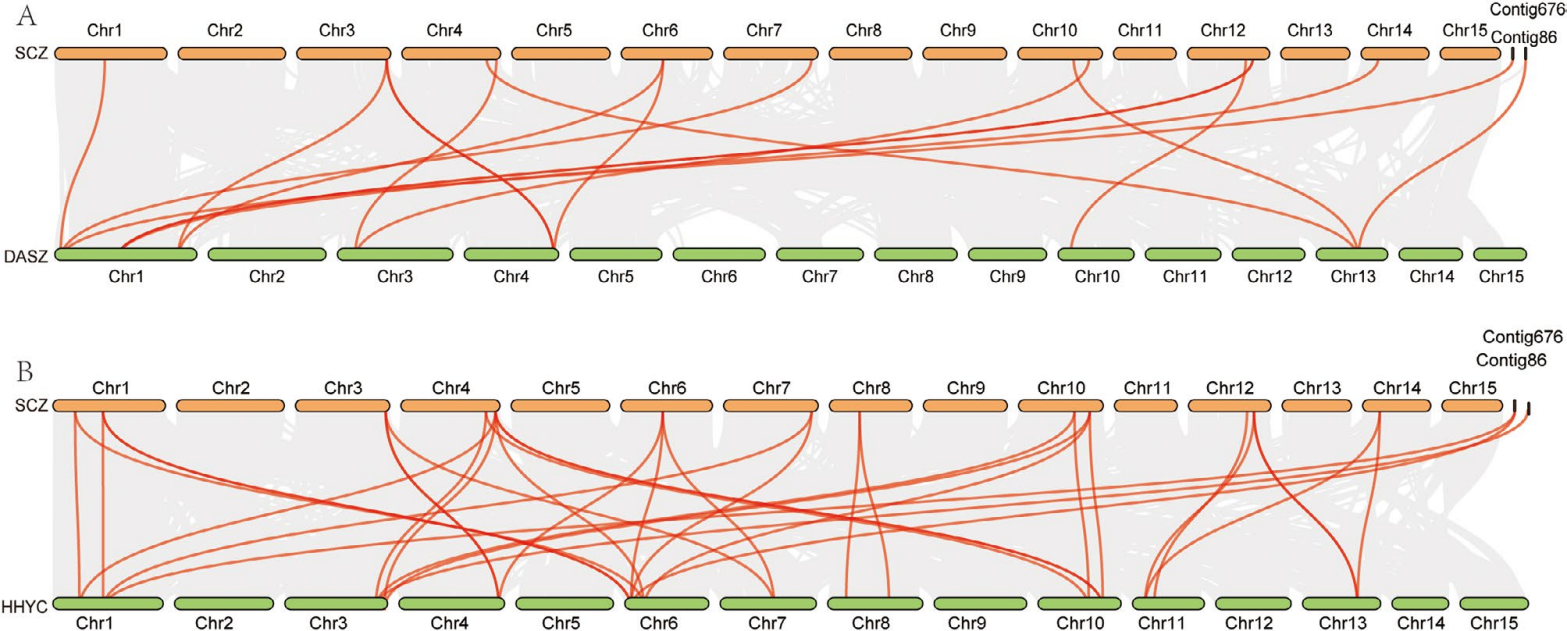
Synteny analysis of PYL genes from SCZ (‘Shuchazao’, *Camellia sinensis*), DASZ (wild tea plant) and HHYC (*Camellia chekiangoleosa*). (A) Synteny analysis between SCZ and DASZ. (B) Synteny analysis between SCZ and HHYC. Red lines connect synteny gene pairs

### Synteny analysis for PYL genes

In the above analysis of PYL gene family sequences, it was found that a large number of PYL genes were highly homologous, suggesting that they may have collinear relationships. Synteny analysis were performed to determine the evolutionary characteristics of the PYL gene family within the SCZ genome. The segmental duplication was found in 9 chromosomes and 2 contig with PYLs gene distribution except for chromosomes 8. A total of sixteen pairs of segmental duplication genes were found within SCZ genome (Fig. 4).

**Fig. 6:**
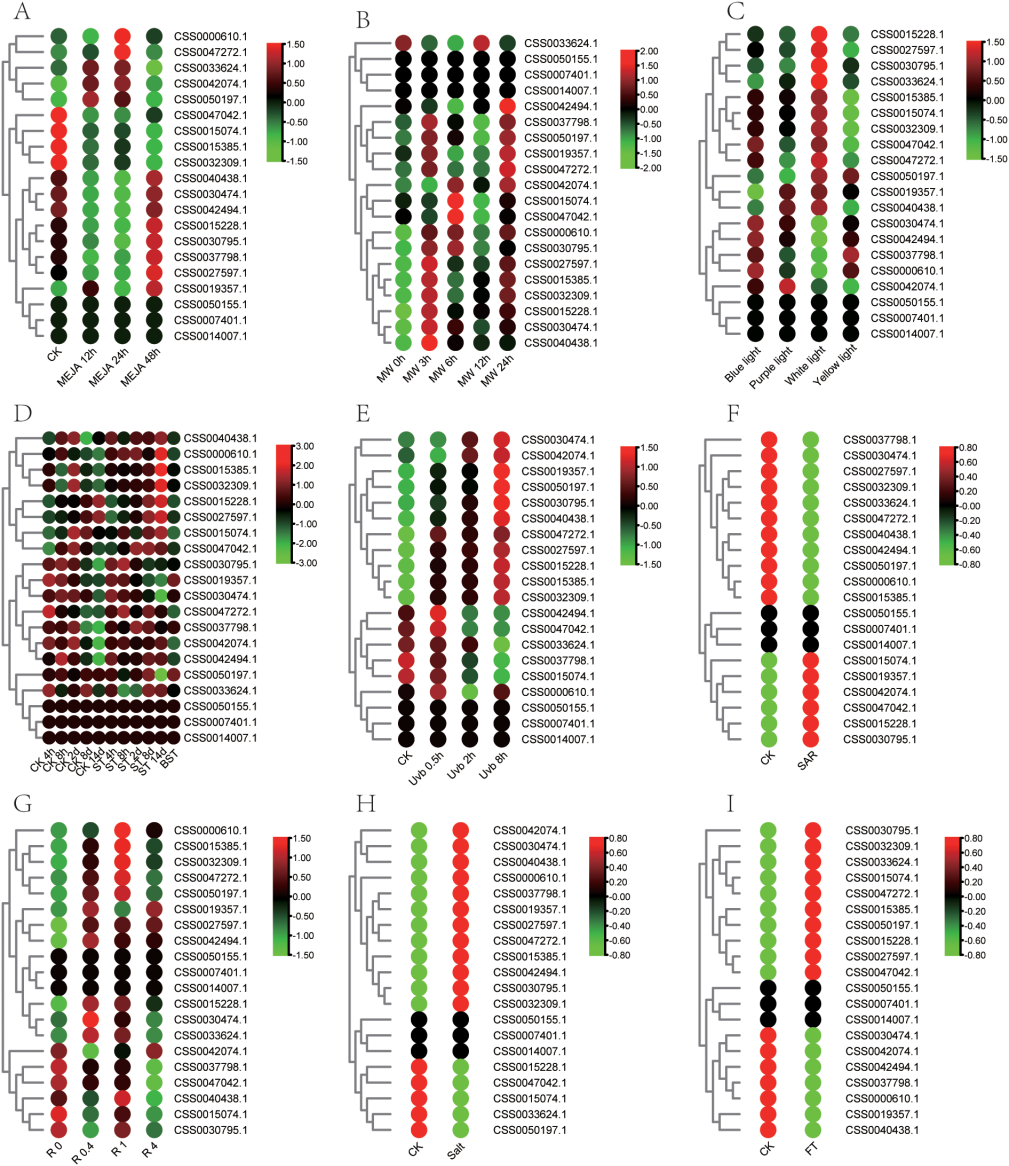
The expression pattern of PYL genes under abiotic stress was analyzed by heat map. (A) Expression pattern of PYL genes under MEJA treatment. (B) Expression pattern of PYL genes under mechanical wound. (C) Expression pattern of PYL genes under different light quality. (D) Expression pattern of PYL genes under shading treatment. (E) Expression pattern of PYL genes under UV treatment. (F) Expression pattern of PYL genes under acid rain. (G) Expression pattern of PYL genes under aluminum treatment with different concentrations. (H) Expression pattern of PYL genes under salt treatment. (I) Expression pattern of PYL genes under fluorine treatment. Data is homogenized by row. Green and red circles indicate lower and higher expression levels, respectively

Notably, all of the identified segmental duplication gene pairs were distributed on different chromosomes except for CSS0042494.1, suggesting that segmental duplication contributed to the expansion of PYL gene family in tea plants. Among them, CSS0007401.1 has the most segmental duplication gene pairs (CSS0015228.1, CSS0027597.1, CSS0017736.1 and CSS0047272.1), while the rest of the genes have one to three segmental duplication events. To further investigate the evolution of PYL gene family, collinearity analysis of SCZ and DASZ, SCZ and HHYC were also conducted (Fig. 5). Interestingly, the results showed that SCZ PYL genes shared 17 and 36 synteny gene pairs with DASZ and HHYC, respectively, which indicated that PYL gene family had a closer genetic relationship in the cultivation of *Camellia* plants, and the PYL gene family in *Camellia chekiangoleosa* experienced a unique expansion event.

**Fig. 7:**
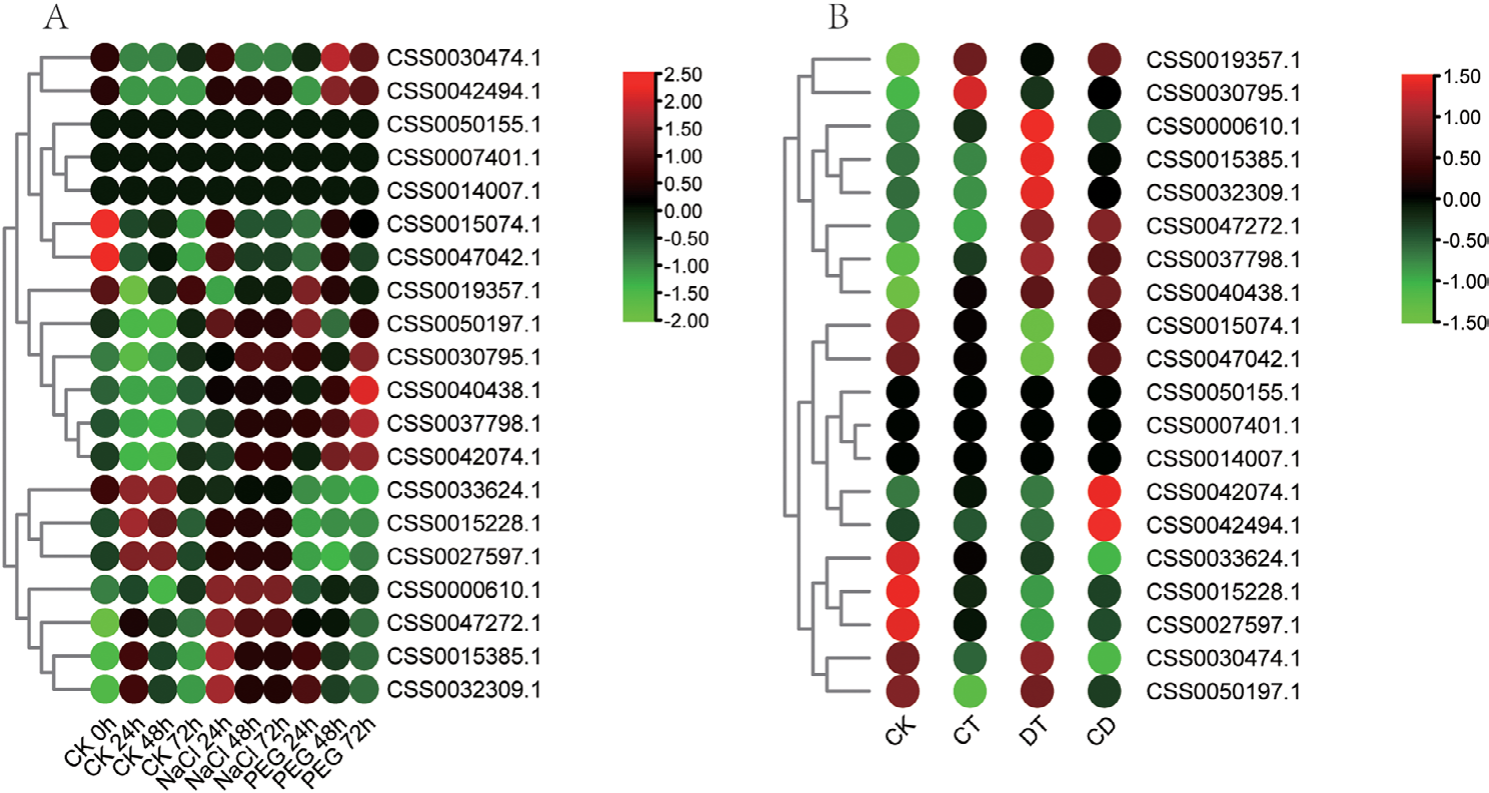
The expression pattern of PYL genes under combined stress was analyzed by heat map. (A) Expression pattern of PYL genes under simulated salt and drought stress. (B) Expression pattern of PYL genes under cold, drought and their combination stress. Data is homogenized by row. Green and red circles indicate lower and higher expression levels, respectively

### Expression analysis of the PYL gene family

In order to explore the role of PYL gene in tea plant response to stress, transcriptome data of various biological and abiotic stresses were collected to identify changes in their expression patterns under these different stresses. The expressions of CSS0033624.1, CSS0042074.1 and CSS0050197.1 genes were significantly up-regulated after 12 and 24 h treatment with MEJA, and the expression of CSS0033624.1 was up-regulated by 4.3 times after 24 h MEJA treatment (Fig. 6A, Table S5). Interestingly, MEJA responsiveness cis-elements were identified in the promoter regions of these three genes (Fig. 3C), indicating that these genes were regulated by MEJA. In addition, the wound-responsive element was also found in the promoter region of CSS0042494.1, and correspondingly, the expression level of this gene was significantly up-regulated after 24 h treatment of mechanical wounding (Fig. 6B). The analysis of light-related transcriptome data showed that the expression levels of four genes CSS0015228.1, CSS0027597.1, CSS0030795.1 and CSS0033624.1 were higher under white light (Fig. 6C). After shading, the expression levels of CSS0015385.1 were significantly up-regulated at 14d, while the expression of CSS0030474.1 was down-regulated (Fig. 6D). The expression level of CSS0015228.1 and CSS0027597.1 were significantly induced by UV (Fig. 6E). Their expression levels increased with the time of UV treatment, and after 8 h of UV treatment, the expression levels of CSS0015228.1 and CSS0027597.1 were 41.6 and 37.7 times of that of the control, respectively. PYL genes family showed three expression patterns after acid rain treatment (Fig. 6F). The first type was up-regulated expression after acid rain treatment, such as CSS0030795.1; the second type is down-regulated expression after acid rain treatment, such as CSS0000610.1; the third type is no significant change after acid rain treatment, such as CSS0050155.1. Furthermore, CSS0042494.1 gene was significantly upregulated by more than 10 times in tea roots treated with different aluminum concentrations (Fig. 6G), suggesting that this gene may play an important role in the process of aluminum stress. PYL genes family also showed three expression patterns after salt stress and fluorine treatment (Fig. 6H and I, Table S5).

**Fig. 8:**
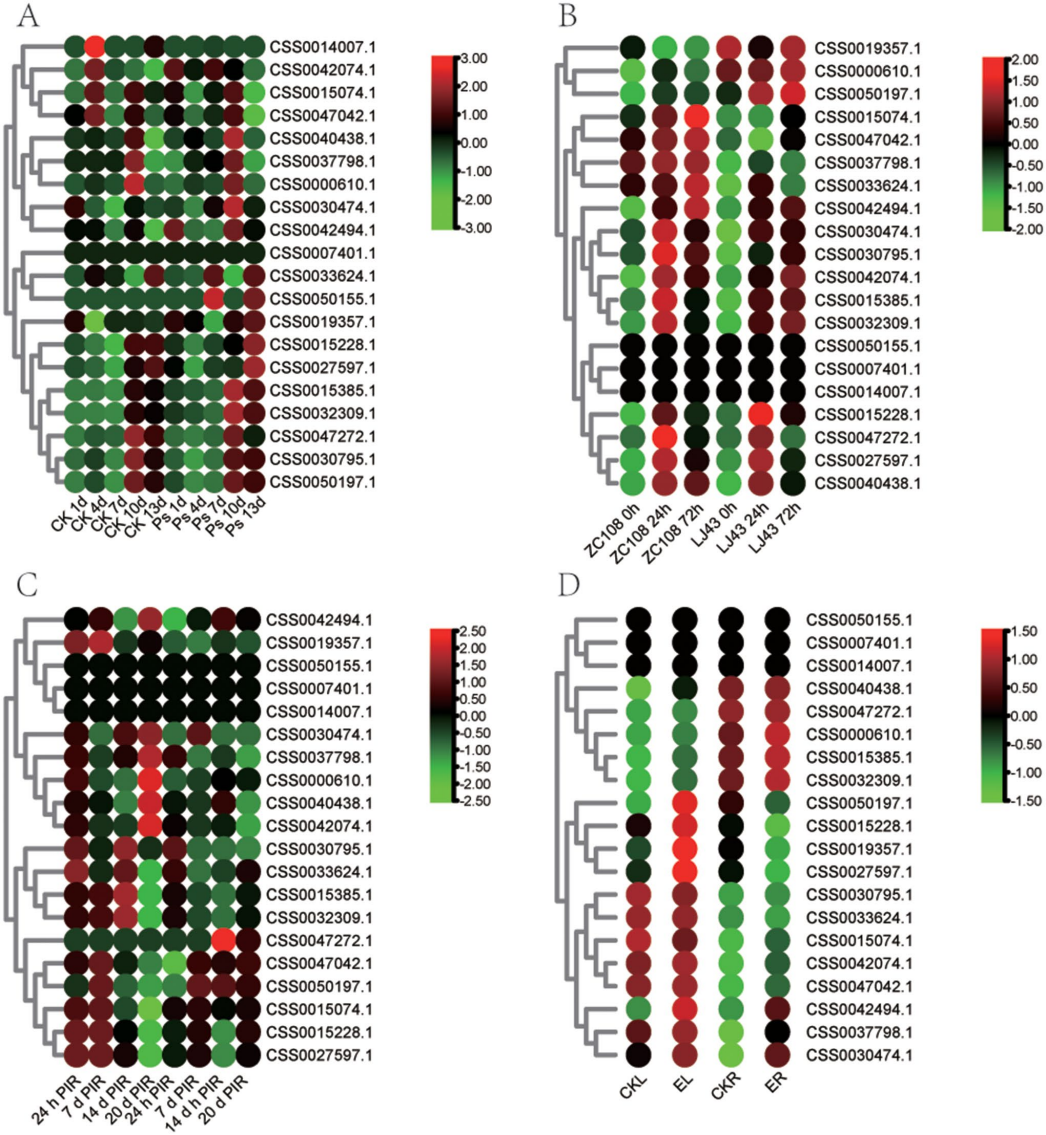
The expression pattern of PYL genes under biotic stress was analyzed by heat map. (A) Expression pattern of PYL genes in pathogens treated with gray blight. (B) Expression pattern of PYL genes in pathogens treated with anthracnose disease. (C) Expression pattern of PYL genes in pathogens treated with blister blight disease (D) Expression pattern of PYL genes under tea geometrid feeding treatment. Data is homogenized by row. Green and red circles indicate lower and higher expression levels, respectively

The transcriptome of the two kinds of stress and the combination of stress were also collected to analyze PYLs gene expression. The expression level of CSS0037798.1 was significantly induced after sodium chloride (NaCl) simulated salt stress and Polyethylene glycol (PEG) simulated drought stress. While, the expression level of CSS0000610.1 gene was induced only by sodium chloride treatment (Fig. 7A, Table S5). Some PYL genes were down-regulated at low temperature, such as CSS0015228.1, CSS0027597.1 and CSS0050197.1. CSS0047272.1 were up-regulated by drought stress, which was 5.4 times that of the control (Fig. 7B). CSS0042074.1 and CSS0042494.1 were upregulated only under combined stress of low temperature and drought stress. CSS0040438.1 can be induced by low temperature, drought and combined stress of low temperature and drought stress, suggesting that it plays an important role in the two stresses.

**Fig. 9:**
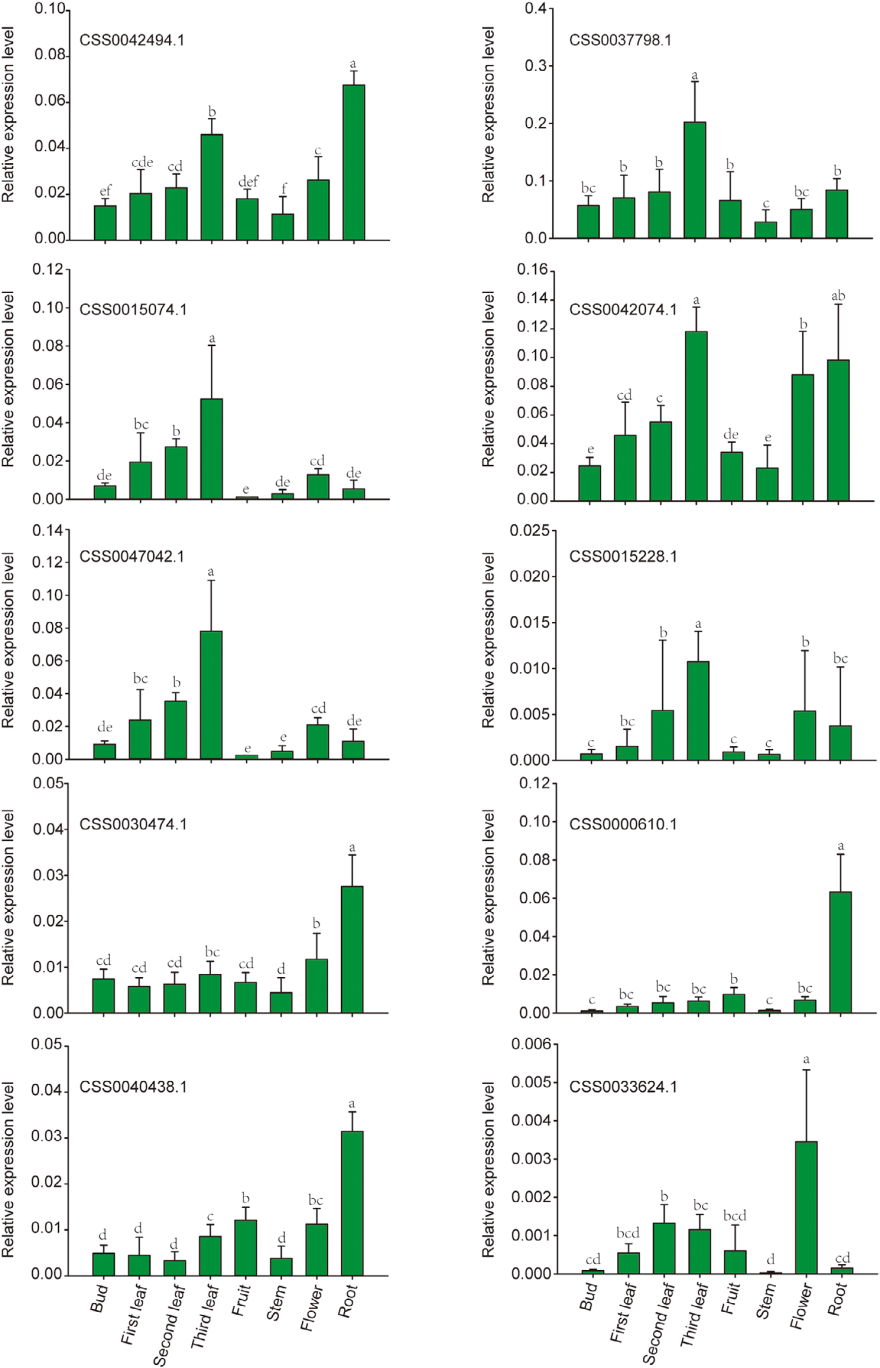
Expression of PYL genes analyzed by RT-qPCR in different tissues of tea plant. Different letters above the bars represent significant differences at p < 0.05. The bars are standard deviations (SD) of three biological replicates

For biotic stress, we collected transcriptome data on three disease infection (gray blight disease, anthracnose disease and tea blister blight) and one pest feeding (tea geometrid) of tea plants (Fig. 8, Table S5). CSS0030474.1 was up-regulated on the 7th and 10th day after gray blight disease infection (Fig. 8A). CSS0015228.1, CSS0027597.1, and CSS0040438.1 were induced by anthracnose disease (Fig. 8B). CSS0050197.1 showed a trend of first increased and then decreased expression after tea blister blight (Fig. 8C). Under tea geometrid feeding treatments, CSS0027597.1 showed the opposite expression pattern in leaves and roots. It was significantly up-regulated in leaves and slightly down-regulated in roots (Fig. 8D). CSS0027597.1 could respond to both anthracnose disease and geometrid feeding treatments, indicating that it plays an important role in tea plants against pests and diseases. All of these results suggest that PYL plays an important role in the resistance of tea plants to abiotic and biotic stresses.

### Verification of PLY gene expression patterns in different tissues by RT-qPCR

PYL genes not only play an important role in stress response, but also participates in the process of growth and development. In order to explore the relationship between PYL gene expression and growth and development of tea plant, 10 PYL genes were selected for expression pattern analysis in 8 different tissues of tea plant by RT-qPCR. The results showed that the expression level of PYL genes were different in different tissues (Fig. 9). The expression levels of seven genes (CSS0042494.1, CSS0037798.1, CSS0015074.1, CSS0042074.1, CSS0047042.1, CSS0015228.1, and CSS0033624.1) in mature leaves were significantly higher than those in tender leaves. For example, the expression of CSS0015228.1 in the third leaf was 15 and 7 times that in the bud and the first leaf, respectively. The expression levels of CSS0015074.1, CSS0042074.1, CSS0047042.1, and CSS0015228.1 genes increased gradually in bud, first leaf, second leaf and third leaf. In addition, CSS0042494.1, CSS0030474.1, CSS0040438.1 and CSS0000610.1 genes were highly expressed in the roots. Among them, the expression level of CSS0000610.1 in the root was significantly higher than that in the other 7 tissues. CSS0033624.1 was highly expressed in flowers, and its expression level was three times or more than that in leaves. The expression levels of CSS0015074.1 and CSS0047042.1 were lowest in fruit. These results indicate that PYL gene plays an important role in the growth and development of tea plants.

## Discussion

In recent years, studies of PYL gene family members have revealed their crucial roles in the growth, development and responses to various stresses of plants [35, 52]. However, these was little information about the PYL proteins in tea plant. Here, we performed a comprehensive analysis on the PYL gene family in tea plant, and identified a total of 20 PYL genes in ‘Shuchazao’ genome. The number of PYL genes have been identified in many plant species, such as rice (Oryza sativa) [53], wheat [54], and grape [55]. Although the number of PYL genes varies among different species, these is no direct relationship was found between the number of PYL genes and the size of genomes. We identified 20 PYL genes in the genome of cultivated tea plant ‘Shuchazao’, while only 11 PYL genes were identified in ancient tea tree using the same method, with the number of the former nearly double that of the latter. Previous studies have found that some gene families, such as OVATE, experienced special expansion events in cultivated tea plants. This result indicates that PYLs may also experience unique expansion events in cultivated tea plants.

Tandem, segmental and whole-genome duplication events of PYL genes have been widely reported in different plant species [56, 57]. For example, among the 14 PYL genes identified in cucumber, there were 5 pairs of collinearity genes [58]. In this study, a total of 20 PYL gene family member were identified in the genome of ‘Shuchazao’. Genome collinearity analysis showed that the PYL members of SCZ and HHYC had more homology, which might indicate that compared with ancient tea plants, PYL genes of cultivated Camellia plants were more preserved or expanded during domestication. In addition, we amplified one of the PYL genes (CSS0047042.1), and found that there was only one amplified band in ‘Shuchazao’ while there were two different amplified bands in ‘Yunkang 10’ (Figure S1). The difference of allelic type indicates that there is extensive genetic diversity between China and Assam tea plants, which may lead to the difference of growth potential between them. As a receptor protein for ABA, PYL plays an important role in plant resistance to stress. In this study, through the analysis of the cis-acting elements in the promoter region of PYL gene in tea plant, a large number of elements related to stress and hormone response were found, suggesting the role of PYL gene in resistance to stress in tea plant. Secondly, through analysis of transcriptome data, we also identified a large number of PYL genes in response to biological and abiotic stress. As an important hormone, Methyl Jasmonate (MEJA) plays an important role in the process of resisting stress in plants. Studies have shown that PYL gene can participate in the stomatal closure of guard cells induced by MEJA [59]. We also found that CSS0033624.1, CSS0042074.1 and CSS0050197.1 could be induced and up-regulated in MeJA-treated transcriptome data, and found MEJA response elements in their promoter regions. This suggests that these three genes may be regulated by MEJA and participate in the process of resistance to stress. The expression of *OsPYL6* gene in rice can affect ABA homeostasis and enhance drought resistance by reducing transpiration to resist dehydration [60]. We found that CSS0040438.1 can be induced by drought stress. In addition to being induced by drought, it can also be induced by low temperature stress, and it can be expressed at a high level under both low temperature and drought stress. These results suggesting that it plays an important role in the two abiotic stresses. In the identification of PYL gene family, it was found that PYL could respond to abiotic stress such as rice [56] and cucumber [58], but there were few studies on PYL gene family under biological stress. In this study, the expression pattern of PYL gene family under biological stress was analyzed, and it was found that CSS0027597.1 could respond to both anthracnose disease and geometrid feeding treatments. This provided the basis for studying the role of PYL in resisting biological stress of tea plant.

In addition to its important role in resisting stress, PYL is also involved in plant growth and development. For example, The ABA receptor *VvPYL1* gene of grape was found to regulate root hair development in transgenic *Arabidopsis thaliana* [33] and *AtPYL13* can regulate seed germination in *Arabidopsis thaliana* [61]. For tea plants, RT-qPCR results showed that PYL genes were tissue-specific, and four PYL genes (CSS0042494.1, CSS0030474.1, CSS0040438.1 and CSS0000610.1) were highly expressed in the roots. Among them, the expression of CSS0000610.1 in root is significantly higher than that of other tissue, suggesting that they might play a role in the development of roots in tea plant. The extremely low expression levels of CSS0015074.1 and CSS0047042.1 in fruits may play a negative regulatory role in their development. At present, there are still few studies on the hormone content in different tissues of tea plants, which hinders our understanding of the diverse expression levels of PYL gene family members in different tissues of tea plants. The preliminary study showed that the contents of ABA and JA were the highest in the second leaf of tea plant, and then decreased rapidly with the rapid development of leaves [62]. Interestingly, we found that with the development of leaves, the expression levels of four PYL genes gradually increased and reached the highest level in the third leaf, suggesting that these PYL genes may respond to the changes of hormone content in plant leaves through cis-acting elements on promoters and participate in the ABA pathway to regulate the process of leaf senescence.

## Conclusions

In this study, we identified the members of PYL gene family in tea plant for the first time. 20 PYL genes were identified in the ‘Shuchazao’ genome, and these genes divided into five subfamilies. Cis-acting element analysis revealed that there were many hormone and stress responsive elements in PYL gene family. Through the analysis of PYL gene expression levels under various biological and abiotic stresses and RT-qPCR between different tissues, multiple PYL genes responding to stress and tissue specific expression were found. Among them, CSS0047272.1 were up-regulated by drought stress, and CSS0027597.1 could respond to both anthracnose disease and geometrid feeding treatments. In addition, CSS0042494.1, CSS0030474.1, CSS0040438.1 and CSS0000610.1 genes were highly expressed in the roots. All of these results will help to reveal the biological functions of PYL genes, and provide a basis for the breeding of stress-resistant tea plant.

## Electronic supplementary material

Below is the link to the electronic supplementary material.

Supplementary Material 1

Supplementary Material 2

Supplementary Material 3

Supplementary Material 4

Supplementary Material 5

Supplementary Material 6

## Data Availability

The sequencing data used in this study are all obtained from NCBI SRA database, and their detailed accession numbers are shown in table S1.
