# Supplementary material for: Genome-wide identification of the PYL gene family of tea plants (Camellia sinensis) revealed its expression profiles under different stress and tissues
Source: BMC Genomics. 2023 Jun 28;24:362. doi: 10.1186/s12864-023-09464-5 (PMC10304716; doi:10.1186/s12864-023-09464-5)
Supplement: Supplementary file 1 — Supplementary Material 1 [file 12864_2023_9464_MOESM1_ESM.pdf]

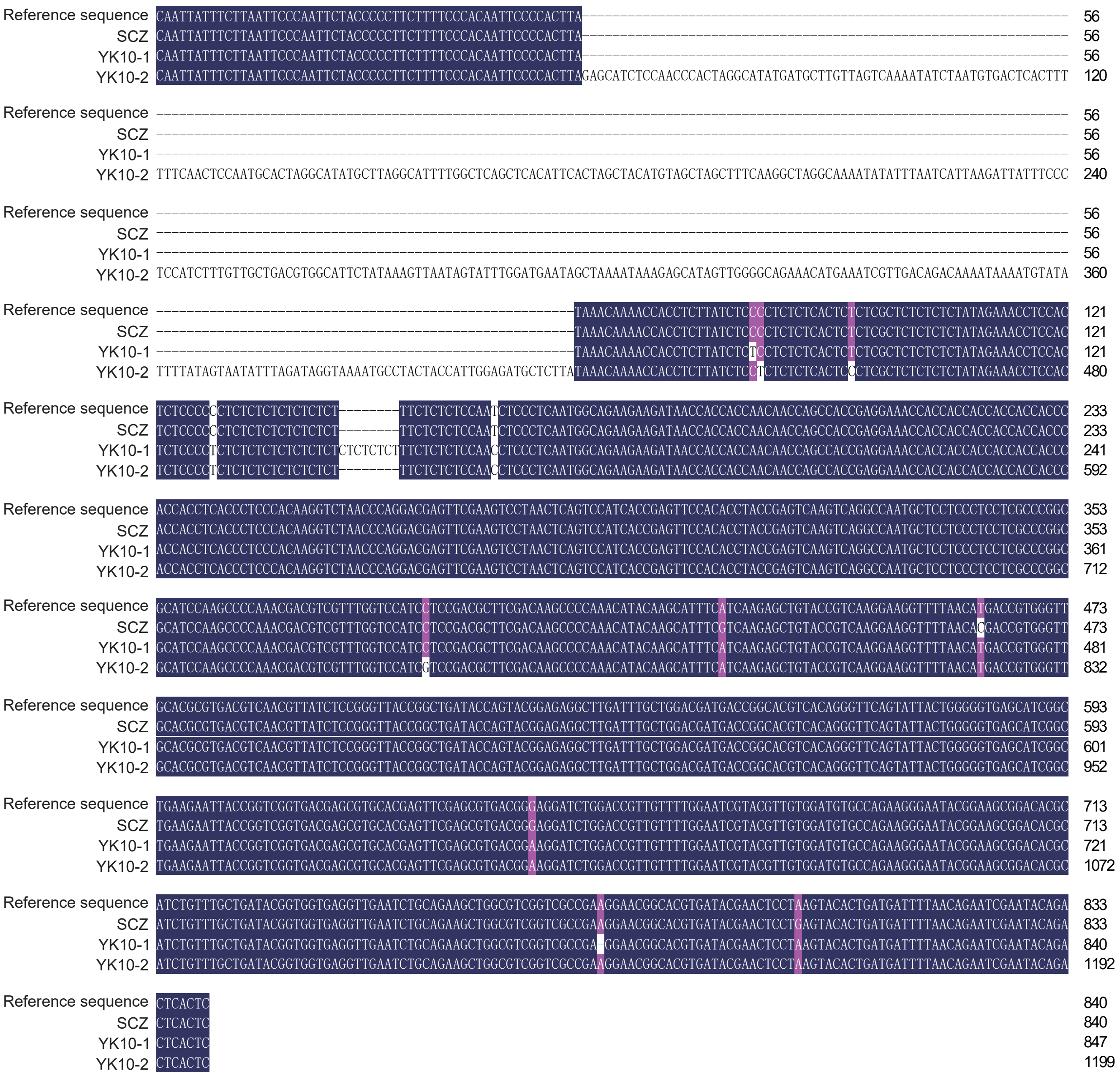

Figure S1. Sequence alignment of CSS0047042.1 gene in reference genome, 'Shuzhazao' and 'Yunkang10'.
